# Action plan co-optimization reveals the parallel encoding of competing reach movements

**DOI:** 10.1038/ncomms8428

**Published:** 2015-07-01

**Authors:** Jason P. Gallivan, Kathryn S. Barton, Craig S. Chapman, Daniel M. Wolpert, J. Randall Flanagan

**Affiliations:** 1 Department of Psychology, Queen’s University, Kingston, Ontario, K7L 3N6, Canada; 2 Centre for Neuroscience Studies, Queen’s University, Kingston, Ontario, K7L 3N6, Canada; 3 Faculty of Physical Education and Recreation, University of Alberta, Alberta, T6G 2H9, Canada; 4 Department of Engineering, University of Cambridge, Cambridge CB2 1PZ, UK

## Abstract

Several influential cognitive theories propose that in situations affording more than one possible target of action, we prepare multiple competing movements before selecting one. Here we provide direct evidence for this provocative but largely untested idea and demonstrate why preparing multiple movements is computationally advantageous. Using a reaching task in which movements are initiated after one of two potential targets is cued, we show that the movement generated for the cued target borrows components of the movement that would have been required for the other, competing target. This interaction can only arise if multiple potential movements are fully specified in advance and we demonstrate that it reduces the time required to launch a given action plan. Our findings suggest that this co-optimization of motor plans is highly automatic and largely occurs outside conscious awareness.

## Abstract (web summary)

Several prominent cognitive theories propose that in situations affording more than one action strategy, the brain prepares multiple competing movements prior to selecting one. Here the authors provide direct experimental evidence for this provocative but largely untested notion.

## Graphical abstract

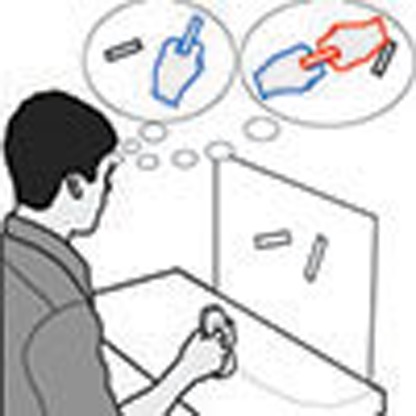

Several highly influential cognitive theories propose that an essential component to perceiving and acting on the world is the simultaneous specification of multiple potential actions afforded by the environment^1^,^2^,^3^. However, direct evidence for this provocative idea is sparse. Recent work has shown that neural activity in brain areas involved in hand actions encodes multiple potential reach targets before deciding between, and then reaching towards, one of these targets^4^. Although this activity might reflect competing movement plans prepared for multiple potential targets^3^, it is well recognized that an equally plausible interpretation is that it instead encodes the sensory properties of the potential targets, such as their visual spatial locations or directions, prior to a single target being selected and the associated movement plan being formed^3^,^5^. Recent behavioural studies have been equally equivocal concerning the motor versus visual encoding of potential reach targets. For instance, it has been shown that when simultaneously presented with multiple potential reach targets and required to act before knowing the final target location, people initially launch their reach movement towards the ‘spatially averaged’ midpoint of the targets before correcting to the chosen target location^6^,^7^,^8^,^9^. While it is certainly possible that this spatial averaging reflects ‘motor averaging’ (that is, the simultaneous implementation of multiple competing single-target motor plans), and is often interpreted as such^6^,^10^,^11^, it is equally consistent with a ‘visual averaging’ across target positions (that is, the preparation of a single movement towards an averaged visual-spatial target location). Recently, using a variant of this ‘go-before-you-know’ task designed to dissociate visual versus motor averaging, we provided evidence suggesting that the initial movement vector reflects an average of multiple competing motor plans rather than an average of visual target locations^12^. Executing an averaged motor plan might be effective in this specific task as is may reduce costs of the movement corrections that are required when the target is cued^9^. However, this does not imply that potential targets are naturally encoded as motor plans in the more common situation in which we select a target (from among alternatives) and then act on it. Determining whether the motor system naturally prepares multiple potential movements when merely presented with alternative targets for action, one of which will subsequently be selected before a movement is even required, is critical to understanding the underlying mechanisms by which the brain initially represents and makes decisions between competing options in the environment.

Some in-roads into tackling this problem may come from considering why—in the first place—the brain might want to prepare multiple competing movements. In general, because tasks can often be achieved using any one of several different actions (for example, a glass can be picked up with the wrist supinated (thumb up) or pronated (thumb down)), movement planning typically involves selecting between these different movement options. Although this redundancy (referred to as the degrees-of-freedom problem in motor control^13^) poses a computational challenge for the motor system, it also provides for flexibility and opportunity. For instance, when presented with multiple competing targets, the motor system could exploit this redundancy by selecting and preparing a set of movement plans—across the potential targets—that share common features or components (for example, the same wrist orientations). This may reduce the requirements of working memory (or improve its resolution^14^,^15^) associated with planning multiple actions, and may allow more rapid movement execution when one of the plans is implemented. By showing that the motor system prepares movements for one target that use the same movement components required for competing targets, we provide direct evidence that the brain, more than merely representing the visual properties of potential targets, fully specifies movements to each potential target.

## Results

**Figure 1:**
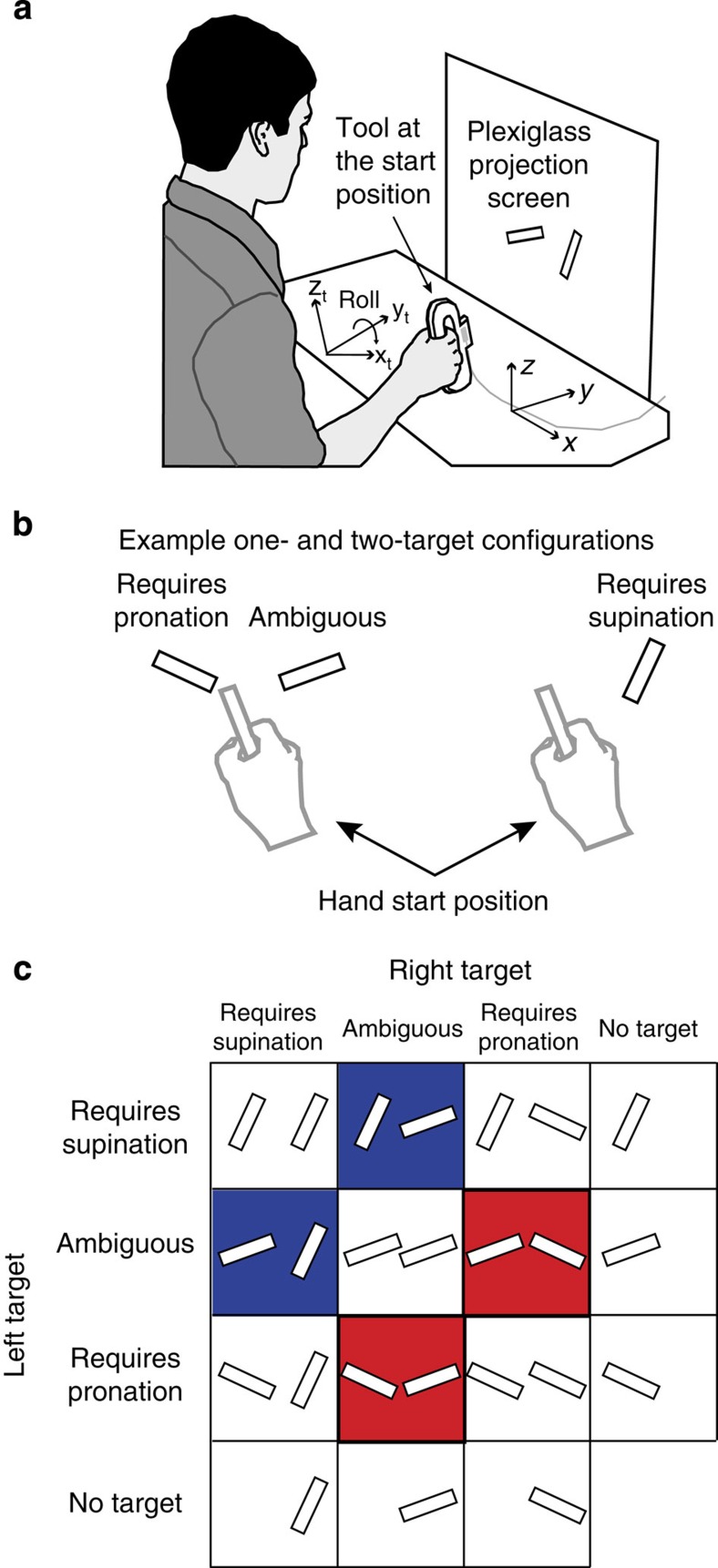
Illustration of experimental set-up and trial types. (**a**) Participants moved the rectangular tip of a hand-held tool to contact one of two potential targets projected onto a vertical screen. (**b**) Example two- and one-target configurations showing the hand and hand-held tool at the start position. (**c**) The nine possible two-target displays and six possible one-target displays. Target displays were made up of three different target types: the supination target requiring a tool-tip angle of 25°, the pronation target requiring a tool-tip angle of −65°, and the ambiguous target that could be comfortably contacted using a tool-tip angle of either 70° (supination) or −110° (pronation). The blue and red boxes highlight key displays in which one of the targets was ambiguous and the other required either supination (blue) or pronation (red).

Participants performed two-target and one-target trials in separate blocks. In each two-target trial, participants were presented with two potential rectangular targets on a vertical Plexiglas screen (see Fig. 1a). After a presentation period (2 s), and concurrent with an auditory beep, one of the potential targets was selected (filled in). This provided the cue for participants to move a hand-held rectangular tool-tip, as quickly and accurately as possible, from the start position to contact the target. Each potential target could be one of three types as defined by its orientation: a target requiring wrist pronation, a target requiring wrist supination, or an ambiguous target that could be contacted using either wrist pronation or supination (see Fig. 1b). One-target trials were identical to the two-target trials except that only a single target was presented. The full set of target configurations across one- and two-target trials is illustrated in Fig. 1c (see Methods for further details).

The ambiguous target, the orientation of which was determined in a separate calibration session (see Methods), was rotated +70° from upright (positive corresponds to clockwise). The pronation target was rotated −65° from upright and the supination target was rotated 25° from upright. Thus, the pronation and supination targets were rotated, relative to the ambiguous target, 45° clockwise and counter-clockwise, respectively. Before each trial began participants were required to orient the tool-tip at the start position so that it was rotated −20° from upright. Thus, the magnitude of the tool-tip rotation required to contact the ambiguous target from the start position was the same regardless of whether the wrist was pronated (90° counter-clockwise) or supinated (90° clockwise). In addition, the magnitude of the rotation required to contact the pronation (45° counter-clockwise) and supination (45° clockwise) targets was the same.

To provide the critical test of whether competing movements to potential targets are fully prepared in advance of target selection, our analysis focused on the two-target trials in which the cued target was ambiguous and the non-cued target was unambiguous, requiring either wrist pronation or supination (see red and blue boxes in Fig. 1c). If participants prepare multiple competing actions based on the movements afforded by the competing targets, and then co-optimize across these plans, they should be more likely to supinate or pronate the wrist for the ambiguous target when the unambiguous target requires wrist supination or pronation, respectively. Conversely, if participants do not prepare multiple plans in advance, but rather plan and execute a single reaching movement after the target is selected, then we would not expect the orientation of the unambiguous target to influence the wrist orientation selected when the ambiguous target is selected.

**Figure 2:**
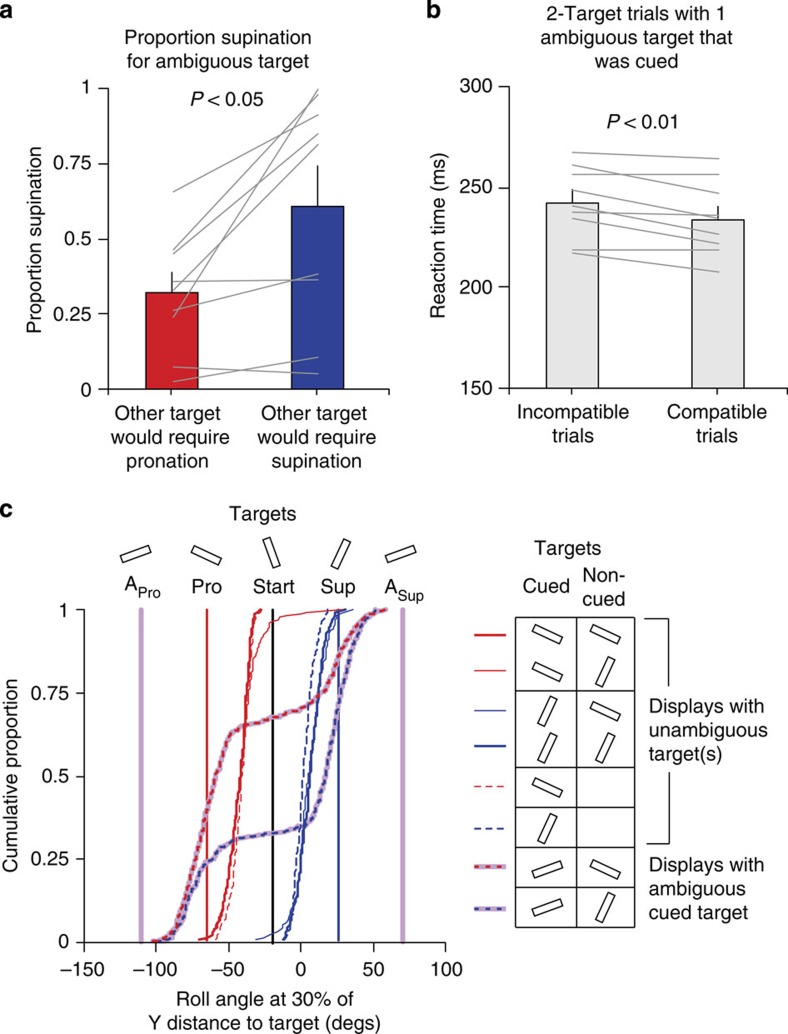
Co-optimization of selected wrist orientation for competing potential targets. (**a**) Bars represent the average proportion, across participants, of trials in which the wrist (and tool-tip) was supinated to contact the ambiguous target in the presence of a competing unambiguous target. This proportion was significantly greater (*t*_8_=3.24; *P*=0.012) when the non-cued, unambiguous target would have required supination (blue) as opposed to pronation (red). (**b**) Bars represent average reaction times, based on participant medians, in two-target trials containing an unambiguous and an ambiguous target. Reaction time was shorter (*t*_8_=3.53; *P*=0.008) when the selected wrist orientation for the ambiguous target was compatible (that is, matched), as opposed to incompatible, with the wrist orientation that would have been required had the unambiguous target been cued. The lines represent proportions from individual participants and error bars represent s.e.m. (**c**) Cumulative distributions of the roll angle at 30% of the Y distance to the target for different trial types (see legend). The vertical dashed lines represent the target wrist orientations.

Figure 2a shows the mean proportion of supination movements (that is, reaches in which the wrist was supinated when contacting the target), averaged across participants, in trials in which the cued (C) target was ambiguous (A) and the non-cued (N) target was either a pronation (P) target (A_C_P_N_ trials) or a supination (S) target (A_C_S_N_ trials). Critically, we found that the proportion of trials with supination was significantly greater (*t*_8_=3.24; *P*=0.012) for the A_C_S_N_ trials (*M*=0.609; s.e.=0.127) than for the A_C_P_N_ trials (*M*=0.321; s.e.=0.066). It is important to note that it is this shift in proportion of wrist supination between A_C_S_N_ and A_C_P_N_ trials that is the effect of interest (that is, the determination of whether participants co-optimize the selection of their movements), and not whether, given the use of a single common ambiguous angle for all participants (as determined from the calibration session), the actual raw proportion of supination values themselves for A_C_S_N_ and A_C_P_N_ trials differed from 0.5.

We also examined whether the proportion of supination trials in A_C_S_N_ and A_C_P_N_ trials was influenced by the wrist orientated selected on the previous trial. A two-way repeated measures ANOVA with trial type (A_C_S_N_ versus A_C_P_N_) and previous wrist orientation (supination versus pronation) failed to reveal a main effect of previous wrist orientation (*P*=0.89) or an interaction between previous wrist orientation and trial type (*P*=0.53). Thus, although previous work on single-target grasping has shown that the wrist orientation selected for an ambiguous target is biased by the orientation selected on the previous trial^16^, we do not see any evidence of such a bias in our considerably more complex task involving reaching towards two targets.

The above result indicates that the movement prepared, but not executed, for the unambiguous target directly influenced the movement prepared, and executed, for the ambiguous target. Importantly, this is despite the fact that the temporal structure of our task—in which target selection (filling in) provides the cue for movement initiation—does not actually require that participants ever consider the non-cued target as a viable option. Notably, no participant, during post-experiment debriefing, reported using an explicit strategy when performing the task. Indeed, had they done so, one might expect that the proportion of trials with supination would be 0 for A_C_P_N_ trials and 1 for A_C_S_N_ trials. Thus, our results suggest that the co-optimization of participants’ movements was highly automatic and occurred largely outside their conscious awareness.

We also examined the mean proportion of supination, averaged across participants, in two-target trials in which both potential targets were ambiguous (A_C_A_N_ trials) and in one-target trials with an ambiguous target (A_C_). We found no significant difference (*t*_8_=0.27; *P*=0.79) in the proportion supination for A_C_A_N_ trials (*M*=0.306; s.e.=0.119) and A_C_ trials (*M*=0.332; s.e.=0.118). This indicates that the ambiguous target, established during one-target calibration, was similarly ambiguous in two-target trials. As expected, we also found that participants consistently pronated and supinated their wrist when pronation and supination potential targets, respectively, were cued as the target. The proportion supination was 0 for all participants in P_C_A_N_, P_C_P_N_, P_C_P_N_ and P_C_ trials, and 1 for all participants in S_C_P_N_, S_C_S_N_ and S_C_ trials. In S_C_A_N_ trials, six participants always supinated but three participants very infrequently pronated (in 7 out of 169 trials combined).

### Reaction time analysis

To assess the influence of different target displays on reaction time (RT), we carried out a set of paired *t*-tests based on participant medians. We used participant medians rather than means to guard against the possible influence of outliers. However, we observed that cumulative RT distributions, collapsing across all target displays, were similar for all participants and approximately normal. Importantly, these paired *t*-tests, which were planned in advance, evaluated independent sources of variance and therefore we did not apply corrections for multiple comparisons.

Overall, the RT in one-target trials (*M*=210 ms; s.e.=21 ms) was significantly shorter (*t*_8_=6.91; *P*<0.001) than in two-target trials (*M*=233 ms; s.e.=20 ms). This finding is in line with several previous studies documenting an RT advantage when planning reach movements towards single targets versus multiple potential targets^6^,^17^,^18^,^19^. For two-target trials involving unambiguous targets, we tested whether RT differed between trials in which the two potential targets had the same orientation (P_C_P_N_ and S_C_S_N_ trials) versus trials in which they did not have the same orientation (P_C_S_N_ and S_C_P_N_ trials). We found that RT in same-orientation trials (*M*=227 ms; s.e.=7 ms) was significantly shorter (*t*_8_=−4.33; *P*=0.002) than that in different-orientation trials (*M*=237 ms; s.e.=7 ms). This RT advantage may arise because in same-orientation trials participants can prepare a component of the to-be-executed movement (that is, wrist orientation) before the target is cued^17^,^20^.

Previous work on reaching to ambiguous targets has shown that when the participant is required to reach as quickly as possible following target presentation, reaction time is greater for ambiguous targets (targets that can be contacted with either wrist pronation or supination) than for unambiguous targets^16^. In the current study, targets were presented 2 s before the movement go signal and therefore, we would not necessarily expect to see an ambiguous target effect on reaction time. Accordingly, for one-target trials, the reaction time in ambiguous (A_C_) trials (*M*=214; s.e.=7 ms) did not statistically differ (*t*_8_=1.81; *P*=0.11) from the reaction time in unambiguous (P_C_ and S_C_) trials (*M*=209; s.e.=7 ms). To assess this same question for two-target trials, we first considered trials presenting one ambiguous potential target and one unambiguous potential target (A_C_P_N_, A_C_S_N_, P_C_A_N_ and S_C_A_N_ trials) and found that reaction time did not depend (*t*_8_=2.01; *P*=0.079) on whether the cued target was ambiguous (*M*=237 ms; s.e.=7 ms) or unambiguous (*M*=233 ms; s.e.=7 ms). We then considered trials in which the two potential targets had the same orientation (A_C_A_N_, P_C_P_N_ and S_C_S_N_ trials) and found that reaction time did not depend (*t*_8_=0.12; *P*=0.91) on whether the two targets were ambiguous (*M*=228 ms; s.e.=8 ms) or unambiguous (*M*=227 ms; s.e.=8 ms).

As noted above, in trials in which the cued target was ambiguous and the other potential target was either a pronation or a supination target, we found that participants were more likely to supinate if the non-cued target required supination (A_C_S_N_ trials) in comparison with when the non-cued target required pronation (A_C_P_N_ trials). However, all nine participants still sometimes pronated in A_C_S_N_ trials and sometimes supinated in A_C_P_N_ trials. Importantly, we could therefore examine how RT was influenced depending on whether the tool-tip angle selected for the ambiguous target was compatible or incompatible with the tool-tip angle required for the non-cued unambiguous target. The participant with the lowest number of incompatible trials still produced nine incompatible trials (∼10%) and thus we decided to include all participants in the analysis (the average percentage of incompatible trials was 34%). Collapsing across A_C_P_N_ and A_C_S_N_ trials, we found that reaction time in compatible trials (*M*=235; s.e.=7 ms) was significantly shorter (*t*_8_=3.53; *P*=0.008) than that in incompatible trials (*M*=243 ms; s.e.=6 ms) (Fig. 2b). Critically, this suggests a RT advantage when competing ambiguous and unambiguous potential targets are encoded as affording a compatible wrist orientation. This result suggests that one reason the motor system preferentially selects a compatible wrist orientation (that is, co-optimizes action plans across targets) is to minimize RT and thereby perform the instructed task more effectively.

As in our RT analysis, we tested whether, in trials in which the cued target was ambiguous and the other potential target was either a pronation or a supination target (A_C_P_N_ and A_C_S_N_ trials), movement duration depends on whether the tool-tip angle selected for the ambiguous target was compatible or incompatible with the tool-tip angle required for the non-cued unambiguous target. We found that movement duration in compatible trials (*M*=207; s.e.=8 ms) was slightly but significantly shorter (*t*_8_=2.42; *P*=0.042) than that in incompatible trials (*M*=216 ms; s.e.=8 ms). As with the RT results, this suggests an MT impairment when the competing ambiguous and unambiguous potential targets are not recognized as affording a shared wrist orientation. Thus, in addition to minimizing RT, the benefits of a reduced MT may further account for the tendency of the motor system to select a compatible over incompatible wrist orientation.

### No evidence for the spatial averaging of action plans

**Figure 3:**
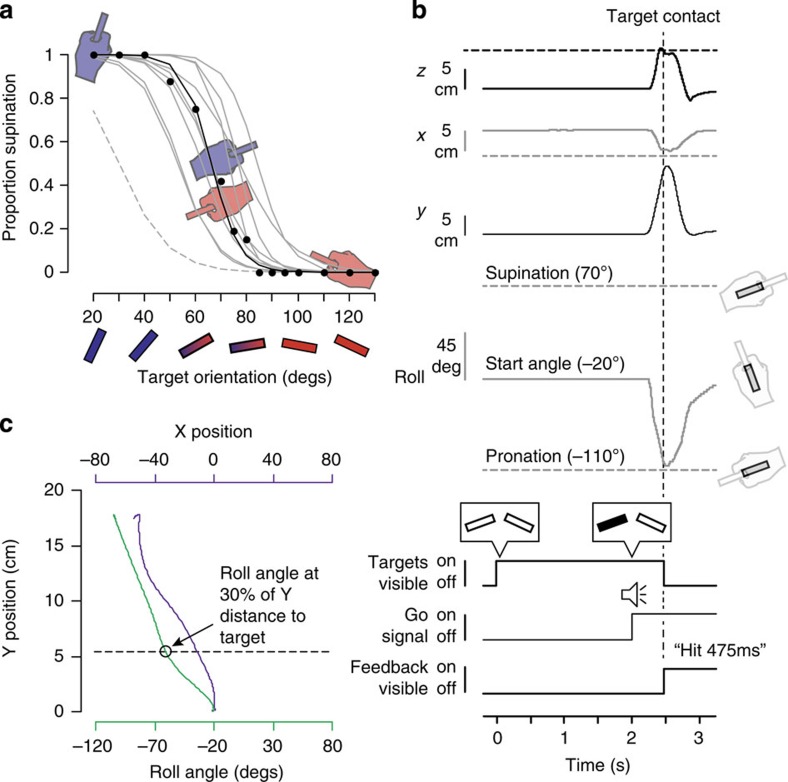
Results from the calibration session and trial sequence. (**a**) The probability of supinating the wrist as a function of target angle (black circles) is shown for a single representative participant together with a logit function (black trace) fitted to the data. The participant always supinated and pronated the wrist for target orientations of 20 and 130°, respectively, but could either supinate or pronate the wrist for the 70° target (see hand drawings). The grey lines show logit functions for all other participants. A target orientation of 70° provided an adequate ambiguous target, eliciting both wrist supination and pronation, for all but one participant (see dashed line at far left). (**b**) Time-varying kinematic variables (top) and corresponding trial events (below) from a single two-target (AS) trial for a representative participant. Following the presentation of potential targets for 2 s, an auditory beep was played concurrently with the final target being selected (filled in); both events provided the cue for subjects to quickly initiate a movement to the filled target. Once the screen was contacted (vertical dashed line), the targets were removed and text feedback was provided on the screen. The horizontal dashed lines represent the target positions and angles. (**c**) X-Y (purple) and roll-Y (green) paths for the same trial shown in **b**. The open circle marks the roll angle when the tool-tip reached 30% of the Y distance to the target (dashed line).

As movements in the current study were initiated only after the final target was cued, we would not expect to observe any ‘spatial averaging’ of reach trajectories as found in previous work that has used go-before-you-know tasks^6^,^9^,^21^,^22^,^23^,^24^. To test for averaging of initial wrist orientation, we examined the roll angle at the time at which the tool-tip reached 30% of the Y distance to the target (see example in Fig. 3c). We will refer to this as the initial roll angle. Figure 2c shows separate cumulative distributions (combining all participants and trials) of the initial roll angle for key one- and two-target trial types. The solid red lines represent trials in which the cued target was the pronation target and the non-cued target was the pronation (thick) or supination (thin) target. The solid blue lines represent trials in which the cued target was the supination target and the non-cued target was the supination (thick) or pronation (thin) target. For comparison, the dashed red and blue lines represent one-target trials with the pronation and supination targets, respectively. The red- and blue-dashed purple lines represent trials in which the cued target was ambiguous and the other competing target was a pronation or supination target, respectively. The red and blue vertical lines represent the target wrist angles for the pronation and supination targets, respectively. The purple vertical lines represent the target wrist angles for the ambiguous target when selecting pronation (left line) and supination (right line), respectively.

If participants’ initial wrist orientations were averaged across target orientations then we should find that, in trials with two unambiguous targets, the initial roll angle should depend on whether the orientations of the two targets are the same or different^9^. However, it is evident from Fig. 2c that the distributions of initial roll angles are very similar for these different trial types. A two-way repeated measures ANOVA with cued target (pronation versus supination) and target pairing (same versus different) as factors revealed no effect of pairing (*P*=0.38) on the initial roll angle and no interaction between pairing and cued target (*P*=0.53).

Note that the red- and blue-dashed purple lines nicely illustrate our main finding; in trials in which the ambiguous target is cued, the initial roll angle is most often in the pronation direction when the unambiguous, non-cued target is a pronation target (red-dashed purple line), and the initial roll angle is most often in the supination direction when the unambiguous, non-cued target is a supination target (blue-dashed purple line). As can be directly appreciated by visual inspection of the figure, on average the roll angle in all trial types reached about 50% of the final roll angle (that is, the roll angle at the target) when the tool-tip reach 30% of the Y distance to the target.

We also tested for averaging of initial reach movement direction. For this analysis, we reasoned that if participants’ movement directions were spatially averaged across target positions then we should find that the initial direction of movement in two-target trials is biased towards the spatial midpoint position of the two potential targets^6^,^18^. This would result in the movement paths being more curved, in the horizontal plane, in two-target trials compared with one-target trials. However, we found that an analysis of the average path curvature, based on participant means, revealed no such differences (*t*_8_=0.77; *P*=0.47) between two-target (*M*=0.120; s.e.=0.006) and one-target (*M*=0.116; s.e.=0.006) trials. Moreover, on average the paths for both one- and two- trials were quite straight, as a value of 0.120 (for two-target trials) indicates that the maximum deviation from a straight line was 12% of the straight-line distance to the target. Taken together, the above findings indicate that, in our task, (1) the initial movement did not arise from an average of planned movements and (2) participants did not prepare a single movement towards a visual average of the targets.

## Discussion

Here we provide direct evidence that the brain automatically prepares, in parallel, multiple potential reach actions before selecting and executing one of them. Specifically, we show, using a unique rapid movement task, that when one of two potential targets equally affords wrist pronation or supination, the wrist orientation prepared for this ambiguous target is more likely to be compatible with the wrist orientation required for the other, unambiguous potential target. Moreover, we find that when the action plan implemented for the ambiguous target is compatible with the action plan required for the unambiguous target, there is a reaction time (RT) and movement time (MT) advantage, indicative of impairment when the action plans implemented to the ambiguous and unambiguous targets are in conflict. These findings are particularly notable given that our task did not actually require the preparation of multiple action plans; participants could have just as easily performed the task by waiting until the final target was selected before preparing and then executing a single movement to that target. However, as we show here, by specifying multiple plans in advance, individuals were able to co-optimize these plans so as to improve overall performance (that is, lower reaction time and movement time). Thus, in addition to providing some of the clearest evidence to date that the motor system prepares multiple potential actions, these findings also offer a direct account of why the brain would do so. Specifically, the parallel specification of competing movements allows for redundancies in the actions afforded by competing targets to be computed and exploited, and the movements thereby co-optimized. In this regard, the current work provides a novel addition to previous studies in action planning and control, which has focused on the optimization of costs associated with movements to single targets^25^,^26^,^27^,^28^, as opposed to an optimization of costs across multiple potential movements. Note that by ‘parallel specification’ we simply mean that at some point before target selection, multiple motor plans are specified and maintained in memory. Our results cannot address the question of how these plans develop during the planning period, but do indicate that they interact. We contrast this parallel process with a ‘serial’ process, by which the movement is planned and executed only after the target has been selected.

At the level of processing, the co-optimization of action plans to potential targets likely relies on fairly complex mechanisms. Indeed, whereas visually apparent object-related parameters like spatial location and orientation can be directly accessed from the retina (and early visual system), the compatible wrist orientation for a given target configuration must instead be back-solved by the motor system through evaluating and intelligently integrating the final possible hand orientations (supinate or pronate) afforded by each potential target. Thus, the co-optimization behaviour observed here strongly suggests that what is being encoded during planning are motor representations of potential movements to the targets (that is, motor representations of final hand orientations) rather than visual representations of the targets themselves (that is, visual representations of their orientations).

For eye movements, there is strong neural evidence that multiple competing saccades can be prepared prior to one of those saccades being selected and executed^29^,^30^,^31^,^32^,^33^,^34^,^35^. Given this previous work, why then is it not a foregone conclusion that multiple reach motor plans should be similarly specified in parallel? First, eye and arm movements perform drastically different functions. We perform saccadic eye movements ∼2-3 times per second so as to gather information from the surrounding visual environment and construct internal representations of world. As such, it is perhaps not surprising that the oculomotor system should continuously specify multiple potential eye movements for the most salient competing visual stimuli in our environment. Reaching movements, by contrast, are most often used to alter our environments (for example, contact or move objects), and accordingly, they tend to be much more deliberate and occur far less frequently in everyday behaviour. Furthermore, reaching movements require a plethora of additional mechanisms not required by the oculomotor system, including, but not limited to, task-specific control policies for intelligent feedback control^26^,^28^,^36^,^37^,^38^, internal models for mapping between motor commands and limb dynamics^25^,^39^,^40^, and coordinate transformations for mapping targets from gaze-centred to hand-centred coordinates^41^,^42^. Second, the neural circuitry supporting the oculomotor system differs greatly from the circuitry supporting the limb control system. In the superior colliculus—the subcortical structure in which activity associated with saccades to multiple potential targets has been observed^29^,^30^,^31^,^32^—salient visual targets appear to be directly mapped onto motor responses corresponding with saccades to these targets^43^. Here, it is only the omnipause neurons in the brainstem that prevent collicular motor neurons from generating an eye movement until required^43^. The limb control system, by contrast, does not appear to have this same type of directness in visual-to-motor mapping. For instance, although neural activity associated with multiple potential reach options has been observed in dorsal premotor cortex^4^ and, more recently, primary motor cortex^44^, it is clear that target-related activity in the latter, which provides the main source of descending projections to spinal neurons^45^, does not automatically evoke corresponding movements of the limb. This perhaps explains why gating mechanisms equivalent to those achieved by omnipause neurons in the brainstem have not been found in the limb control system. However, despite these functional, computational and neural differences, the current findings suggest that the parallel specification of multiple potential movements may be a basic mechanism exploited by both the oculomotor system and the limb control system when dealing with competing options in the environment.

Why should the motor system co-optimize the planning of actions across multiple potential targets? One likely possibility, made clear by the results presented here, is that choosing a wrist orientation for the ambiguous target that is compatible with the orientation required for the unambiguous target leads to a reliable RT (and MT) advantage, thus minimizing the time required to contact the cued target. At a more general level, this finding is consistent with previous work documenting an RT advantage for reaching movements when multiple potential targets are presented in the same versus different directions^17^,^20^, a finding which we also replicate, in principle, on our same-versus different-orientation trials. On the basis of previous work, this RT advantage is thought to emerge in cases in which some component of the movement is shared among targets and can be partially specified before movement (for example, wrist orientation or reach direction), thereby limiting the need to compute parameters related to that component in an online manner (that is, during the RT interval). Thus, the implementation of co-optimized movements in the presence of multiple competing targets may reflect, in part, a natural tendency for the motor system to capitalize on the basic mechanisms that underlie RT benefits when targets are perceived as affording same versus different movement components (for example, shared orientations or directions).

Another possible reason for why the motor system might co-optimize action plans across potential targets is to optimize the utilization of working memory resources associated with movement preparation. The limited resource capacity of working memory, whether one adheres to a fixed, independent slot model or resource model (for review, see ref. 15) necessitates that only a very small fraction of the multitude of available actions can be equally considered—with any reasonable degree of precision—at any one given moment in time. As such, it would likely be beneficial for the motor system to flexibly streamline and prioritize the representation of certain possible movements over others. This could be done, for example, by specifying some potential actions as being more optimal for achieving the goals of the task and thus, allocating more working memory resources for those actions versus others. The intelligent shaping of action plans to primarily those that use common movement components (for example, shared wrist orientations) would, in principle, constrain both the number and variability (that is, range of hand postures) of potential movements that must be concurrently held in working memory. According to both slot and resource models^15^, this would presumably facilitate working memory processes in the context of action planning and control.

Despite the reaction (and movement) time advantage, our results show that participants did not always select a compatible wrist orientation for the ambiguous target. This clearly suggests that participants did not implement an explicit cognitive strategy during the task, which would be expected to result in consistent co-optimization behaviour (that is, compatible wrist orientation selection) from trial to trial. In support of this notion, as noted in the Results, no participant, during post-experiment debriefing, reported using a particular strategy during testing. Importantly, this suggests that the co-optimization of reach plans arises from automatic visual-motor processes that are largely outside of participants’ conscious awareness. The fact that co-optimization did not always occur may reflect noise in the neural processes underlying the mapping of the viewed orientation of a target onto the movement(s) afforded by that target or the interactions among competing motor plans.

Much of the current empirical evidence for action ‘affordances’—the ecological notion that objects may automatically potentiate actions they afford^1^—comes from psychological studies showing that when participants make perceptual judgments about real or pictured objects, RT is reduced (or prolonged) when the immediate action afforded by the object is compatible (or incompatible) with either the manual response used to register the judgment, for example refs 46, 47, or an action maintained in working memory, for example ref. 48. The current findings extend this previous work in two important ways. First, they show that RT compatibility effects extend to the actions afforded by multiple competing objects in a visual scene, not just those of a single object as explored previously. This finding unites the predominant behavioural phenomena used in the field of cognitive psychology to explore action affordances (that is, compatibility/congruence effects on RT) with increasingly influential models that view behaviour as a constant competition between internal representations of multiple potential actions; that is, affordance competition, see ref. 2. Second, they show that compatibility effects, rather than being constrained to indirect measures of cognitive processing like RT, can in fact be ‘read-out’ from details of the movement itself (in this case, whether a pronated or supinated wrist orientation is implemented).

Here we demonstrate, using a task in which the cuing of one of two potential targets provides the instruction for participants to initiate a reach towards that target, that the movement towards this selected target often utilizes the same parameters necessary for acting on the competing, unselected target. Notably, this sharing of action parameters across potential movements was not only accompanied by reaction and movement time advantages but also occurred in the complete absence of any spatial averaging behaviour, which to date has been the primary behavioural phenomenon used as supporting (though, not definitive) evidence for the idea that the brain specifies multiple movements in parallel^6^,^9^,^10^,^18^. An interesting avenue for future research is to explore whether, in addition to minimizing reaction and movement time, the co-optimization behaviour observed here also optimizes movement accuracy and variability, and how this phenomenon generalizes to different tasks and contexts.

## Methods

### Participants and general procedure

Eleven right-handed participants (aged 20–23, 4 women), recruited from the Queen’s University undergraduate population, took part in this study after providing written informed consent. A target sample size of 10–12 participants was specified in advance based on previous studies in this area and our expectation that, if the main experimental effect was present, it should be observed in almost all participants. The Queen’s University Research Ethics Board approved the study. All participants were naive with respect to the purpose of the experiment. One participant was removed following the initial calibration session (see below) and did not complete the main experiment. Of the 10 participants who completed the main experiment, one was removed from further analysis (see below).

### Apparatus

Participants sat at a table and held a Plexiglas tool that had a 6 × 1 cm rectangular tool-tip extending from its front (Fig. 1a). The shape of the tool allowed changes in the orientation of the tool-tip to closely match changes in orientation of the wrist. Rectangular targets, the same size as the tool-tip, were rear-projected onto a vertical screen located 15 cm (in the y axis) from the start position of the tool-tip and approximately 40 cm (in the y axis) from the participant’s eyes. The screen was covered in Plexiglas and could be contacted forcefully with the tool-tip. Tool-tip position and orientation information was recorded in three dimensions at 240 sample/s using an electromagnetic position sensor (Polhemus Liberty, Burlington, VT) embedded in the tool-tip.

The start position of the tool-tip and the participant’s mid-sagittal plane were aligned with the screen midline (Fig. 1a). Orientations of the tool-tip and targets were defined as 0° when the rectangle’s long axis was vertical with clockwise rotation (from the participant’s perspective) defined as positive. Targets were presented at a height of 15 cm above the table surface (*z*-axis) either 6.5 cm to the left or 6.5 cm to the right of screen midline, or at both locations depending on the trial type.

### Calibration procedure

The aim of the calibration session was to determine the ambiguous target orientation at which, on the whole, participants would be equally likely to contact the target by pronating or supinating the wrist and hand-held tool-tip. In this initial calibration session, participants were presented with single rectangular targets at the following orientations: 20, 30, 40, 50, 60, 70, 75, 80, 85, 90, 95, 100, 110, 120 and 130°. The numbers of trials associated with these orientations were 10, 10, 10, 10, 10, 20, 20, 24, 34, 34, 24, 20, 10, 10 and 10, respectively. Targets were randomly presented at the left or right target locations (256 trials in total). Larger numbers of trials were included in the middle part of the range of angles because we anticipated that the switch between pronation and supination wrist orientations would occur in this region^16^ and we wanted to ensure adequate detection of this switch point in each participant.

At the start of each trial in the calibration session, the participant was required to hold the tool at the start position with the tool-tip oriented vertically (0°) and the bottom edge of the tool resting on the table. A thin (2 mm) piece of wood attached to the table helped participants position the tool at the start position. Once the tool-tip was held within 1.25 cm of the start position for 200 ms, a single target was presented for 750 ms followed by a beep (100 ms, 1,000 Hz) that cued the participant to reach towards and contact the target, aligning the position and orientation of the tool tip with the target.

The circles in Fig. 3a shows, for a single participant, the proportion of trials in which supination was employed as a function of target orientation. To determine the point of subjective equality (PSE), at which the participant would be equally likely to use pronation or supination, we fit the following logit function to the data:




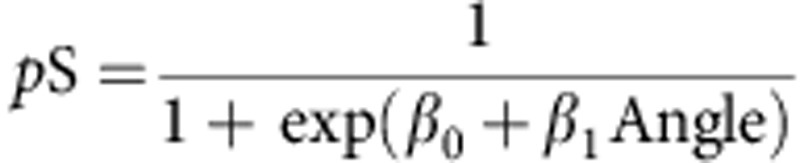




where *p*S denotes the probability of supination (black line in Fig. 1a). The PSE is given by -β_0_/β_1_ and was 66.6° for this participant. The grey lines in Fig. 3a show the logit functions for the other 10 participants. One participant was clearly an outlier (see dashed line) and was not tested in the main experiment. The average PSE of the remaining 10 participants was 68.0° (s.e.=10.7°). Therefore, we selected an angle of 70° as our ambiguous target angle for the main experiment. One of these 10 participants always supinated when contacting the ambiguous target in the main experiment and thus, their data could not be used to evaluate the hypothesis being tested. Perhaps not surprisingly, this participant, who was removed from the analysis, exhibited the largest PSE in the calibration session (see most rightward curve in Fig. 3a).

### Experimental procedure

In the main experiment, all participants first performed 340 two-target trials. There were nine different two-target displays (Fig. 1c) involving different combinations of the three target types: ambiguous (A), unambiguous pronation (P) and unambiguous supination (S). There were 60 trials for each of the four displays that included the ambiguous target on either the left or right and either the pronation or supination target on the opposite side of the display (see red and blue boxes in Fig. 1c), and 20 trials for each of the remaining five displays. For each display, each target had an equal probability of being cued. The order of trials, each with a particular target display and cued target, was fully randomized.

At the beginning of each trial, a start target (equal in size to the tool-tip) oriented at -20° was displayed on the screen, midway between the left and right potential target locations and 10 cm below (see Fig. 1b which shows the tool at the start position). In addition, a rectangle (equal in size to the start target) representing the projection of the tool-tip onto the screen was displayed and the participant had to align the projected tool-tip with the start target while holding the tool-tip 15 cm from the screen (*y* axis). The colour of the projected tool-tip indicated whether the tool-tip was held within 2 cm and 5° of the start position (green) or not (red). With this feedback, participants could easily and quickly position the tool-tip in the required start position. Once the tool-tip was held in the correct position for 1.5 s, the start target disappeared and the two potential targets (unfilled rectangles) were presented (see Fig. 3c, which shows time-varying kinematic variables and corresponding trial events from a single two-target (A_C_P_U_) trial for a representative participant). After a brief delay of 2 s, one of the potential targets was cued as the target (filled in) and a brief auditory tone (100 ms, 1000 Hz) sounded, together providing a go signal instructing the participant to initiate a movement to contact the target. After the screen was contacted, the time to contact the screen (that is, the time from the go signal to screen contact) and whether the trial was a ‘hit’ or a ‘miss’ was displayed centrally on the screen. The trial was considered to be a ‘hit’ if the centre of the tool-tip was within 2 cm of the centre of the target and the orientation of the tool-tip was within 15° of the orientation of the target. Finally, if the participant initiated the movement <100 ms after the go signal (that is, before the movement could have been triggered by the go signal), the targets were removed from the screen, the message ‘too early’ was displayed, and the trial was re-run later in the session. The experimenter instructed participants move quickly and accurately. However, there was no explicit movement time requirement and participants did not receive feedback indicating if a given movement was too slow (or too fast).

Following the completion of these two-target trials, participants then completed 60 one-target trials, each with the same timing as the two-target trials. There were 10 trials for each of six different target displays (Fig. 1c) with one of the three target types (ambiguous, pronation and supination) located at either the left or right target position. Trial order was fully randomized.

### Data analysis

For each trial, we determined the roll angle (Fig. 1a) of the tool-tip at the time the tool-tip contacted the screen. The wrist was classified as having been supinated if the roll angle at contact was greater than −20° (that is, if the wrist rotated clockwise from the start angle) and as having been pronated if the roll angle was less than −20° (that is, if the wrist rotated counter-clockwise from its start angle). To evaluate the change in roll angle during the initial component of the movement, we also determined the roll angle when the tool-tip reached 30% of the Y distance to the target (see Fig. 3c that shows X-Y and roll-Y paths for the trial illustrated in Fig. 3b). For each trial we also recorded the reaction time and the movement time as defined above. Paired *t*-tests, with a *P*-value of 0.05, were used to compare dependent measures across conditions.

### Hit frequency and trial selection

The percentage of successful hits (versus misses) ranged from 82 to 98 percent across the nine participants (*M*=91; s.e.=6) and we observed no difference between two-target and one-target trials (*t*_8_=1.041; *P*=0.328). In all of the analyses reported in the remainder of the results, only hit trials were included. To ensure that movements were not initiated before the Go signal, and to avoid long reaction times, we excluded trials with reaction times that did not fall between 150 and 350 ms. This resulted in the removal of 54 out of a total of 3,590 successful hit trials (1.5 percent).

## Additional information

**How to cite this article:** Gallivan, J.P. *et al.* Action plan co-optimization reveals the parallel encoding of competing reach movements. *Nat. Commun.* 6:7428 doi: 10.1038/ncomms8428 (2015).
